# Assessing the Policy Implications of Different Definitions for Added Sugars: An Analysis Across the Australian Packaged Food and Beverage Supply

**DOI:** 10.1016/j.cdnut.2023.102058

**Published:** 2024-02-20

**Authors:** Daisy H Coyle, Tazman Davies, Fraser Taylor, Kylie Howes, Simone Pettigrew, Alexandra Jones

**Affiliations:** Faculty of Medicine, The George Institute for Global Health, University of New South Wales, Sydney, NSW, Australia

**Keywords:** Added sugar, food labeling, food policy, packaged food, population health, public health nutrition

## Abstract

**Background:**

In recent years, various definitions of "added sugars" have emerged across jurisdictions. Although it is clear how these definitions differ, there is limited understanding of the policy implications associated with these variations.

**Objective:**

To test the potential policy implications of different definitions of “added sugars” on the Australian packaged food supply, we developed a method to estimate the content of “added sugars” in packaged foods and applied this to 3 different definitions of “added sugars”: (i) United States Food and Drug Administration (US FDA) added sugar definition, (ii) the World Health Organization (WHO) free sugar definition, and (iii) a comprehensive definition that was developed from a review of the evidence on “added sugars.”

**Methods:**

Using a representative sample of 25,323 Australian packaged foods, the “added sugar” content and proportion of products that contain “added sugar” under the 3 definitions were estimated. In addition, a comparative analysis exploring the impact of the US FDA definition (least comprehensive) vs. the comprehensive definition was conducted to understand potential implications of adopting different regulatory definitions in Australia.

**Results:**

The US FDA definition identified the lowest number and proportion of products with any “added sugars” at 14,380 products (representing 56.8% of all products), followed by the WHO free sugar definition at 15,168 products (59.9%) and the comprehensive definition at 16,260 products (64.2%). The mean estimates for “added sugars” were 8.5 g/100 g, 8.7 g/100 g, and 9.6 g/100 g for the US FDA, WHO, and comprehensive definitions, respectively. Compared with the US FDA definition, the comprehensive definition captured an additional 7.4% of products, largely driven by nonalcoholic beverages, special foods and fruit, vegetables, nuts, and legumes.

**Conclusions:**

Despite small variations in different “added sugars” definitions, their application has some significant policy implications. Findings highlight the importance of applying a comprehensive regulatory definition that adequately captures all sugars that have been linked to poor health.

## Introduction

High sugar intakes can contribute to a wide range of unfavorable health outcomes including unhealthy weight gain [1,2], dental caries [3], and an increased risk of noncommunicable diseases, such as type 2 diabetes [3], cardiovascular disease [4,5], stroke [4], and certain cancers [[6], [7], [8]]. Sugars can occur naturally in foods such as fruit and dairy products, and can be added to foods and beverages by manufacturers during processing and manufacturing for flavor, texture, and preservation purposes. Sugars can also be added by consumers during cooking and food preparation. In Australia, the context of the present study, sugars added by manufacturers and consumers together account for approximately 57% of total sugar intake in the average diet [9]. This is primarily driven by the consumption of energy-dense, nutrient poor discretionary foods including confectionary, sugar sweetened beverages, and ice creams [10].

Governments have shown increasing interest in designing policies to reduce excess sugar intake, including through changes to food labeling such as requirements to quantify “added sugars” in nutrition panels, or implementation of front-of-pack warning labels to alert consumers about products that contain excess “added sugar” levels [11,12]. While there is broad consistency in many of the sugars targeted by these policies, there remain variations in the definitions developed and implemented for different uses across jurisdictions. For instance, the WHO uses the term “free sugars,” which refers to sugars that are not contained within the cell wall of a food and includes sugars added to foods and drinks, as well as sugars naturally occurring in honey, syrup, and fruit juice [13]. Conversely, the United States Food and Drug Administration uses the term “added sugar,” which refers to sugars added during the processing of foods as well as syrups, honey, and concentrated fruit and vegetable juices [11].

The Australian Government is currently considering a definition of “added sugars” for use in food labeling and nutrition claim policies, among other potential reforms [14,15]. The definition needs to be evidence-informed and reflect current research regarding the health impact of different types of sugars [16]. It is also important that this definition is future-proofed to minimize ambiguity and reduce the risk of loopholes that may arise from the use of novel ingredients as substitutes for traditional sugars. The objective of this study was to develop a method for estimating the content of “added sugars” in packaged foods to test the practical implications of different “added sugars” definitions for Australia. These definitions were (i) the United States Department of Agriculture (US FDA) added sugar definition, (ii) the WHO free sugar definition, and (iii) a comprehensive definition developed from a comparative analysis of evidence of definitions for “added” and “free” sugars used in law and policy around the world mapped against current available health evidence on these sugars [16]. Under these 3 scenarios, we assessed the quantity and percentage of products in the Australian food supply containing “added” or “free” sugars. We also estimated the “added sugar” content of packaged foods across each of the 3 definitions and compared this against the total sugar content. To illustrate the potential implications of adopting different definitions into policy, we conducted a secondary analysis comparing the US FDA definition (least comprehensive) against the comprehensive definition to quantify the disparities between the 2.

## Methods

For the purpose of this article, the term “added sugars” relates to both “added sugars” and “free sugars” as stated in the assessed US FDA, WHO, and comprehensive definitions. As this study used secondary nutrition composition data, ethics approval was not required.

### Packaged food and beverage data

To assess the impact of applying the different “added sugar” definitions across the Australian packaged food supply, we used data from the 2022 FoodSwitch database [[17], [18], [19]]. This database contains 35,645 barcoded products available for sale from 5 large supermarket retailers in Australia, representing approximately 97% of packaged products purchased in Australia [20]. The data are collected during in-store collections whereby trained data collectors take photos of all food products available for sale. Information about the nutritional composition (per 100 g and per serve), ingredient information, front-of-pack labeling and claims information is extracted from the photos and entered into the FoodSwitch database as described previously [17].

### Product categorization and exclusion criteria

Products were categorized into 20 major categories based on the hierarchical system developed by the Global Food Monitoring Group and incorporated into FoodSwitch [20]. This system categorizes foods across major categories (e.g., bread and bakery products), categories (e.g., bread), and subcategories (e.g., pita bread). We excluded products that are not required to provide a nutrition information panel (NIP), as they would not be required to display “added sugar” information. These products included alcoholic beverages, coffee and tea, plain water, vitamins and minerals, baby formula, fresh and chilled seafood/meat/poultry, fresh unpackaged bread, products in small pack sizes (e.g., chewing gum), and herbs and spices.

### “Added” and “free sugar” definitions tested

**FIGURE 1:**
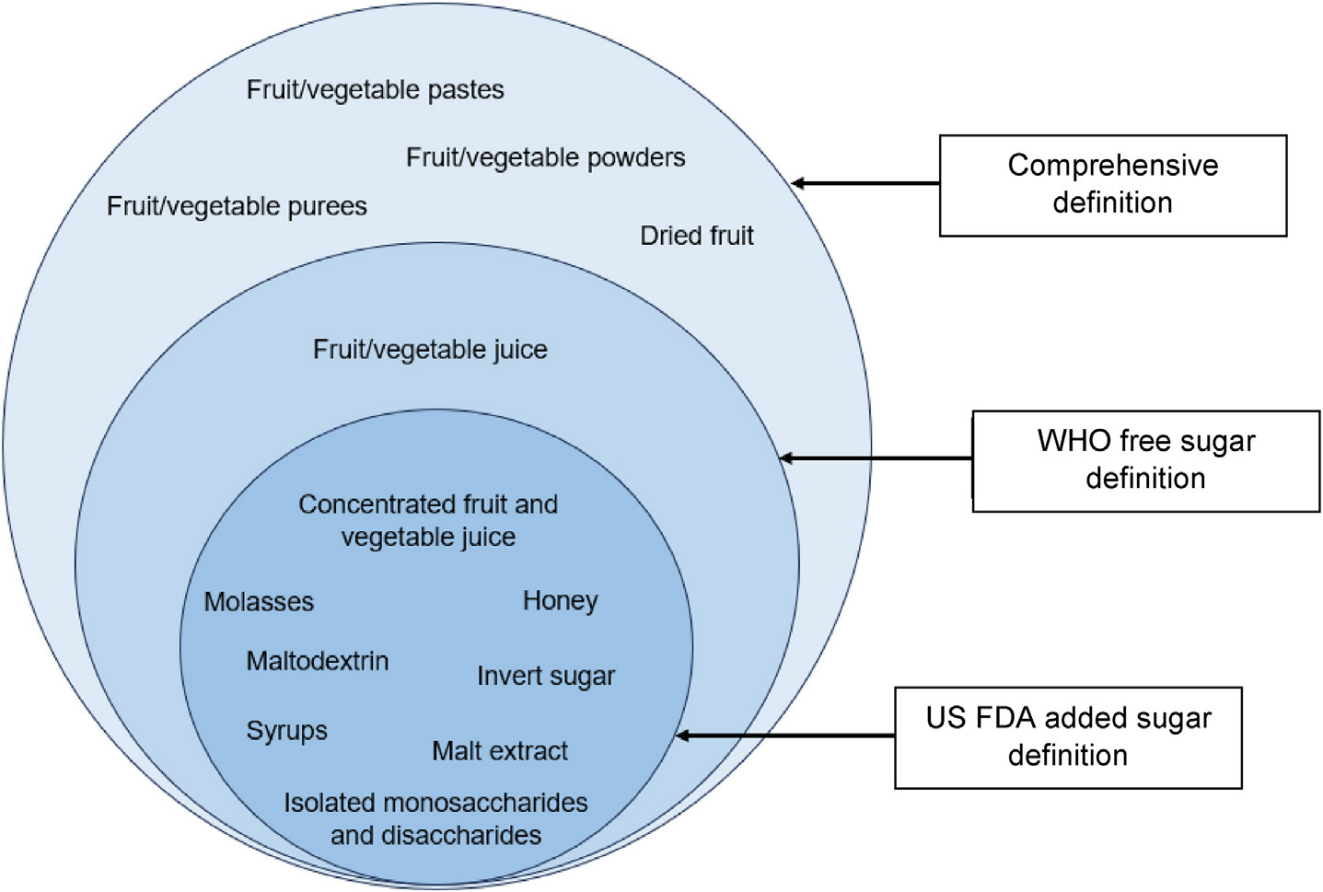
An illustrative example of the types of sugars included in the different definitions of added and free sugars tested.

Figure 1 provides an illustrative example of the types of sugars covered under each of the 3 definitions. The comprehensive definition was developed by researchers who conducted a comparative analysis of a wide range of definitions of “added” and “free” sugars used in law and policy, and mapped these definitions against available evidence on the relationship between specific sugars and ill health to make recommendations on what sugars should be the focus of labeling reform [16]. This definition was considered the gold standard of the 3 definitions tested given its inclusion of all “added” and “free” sugars that have been linked to poor health outcomes. A full list of food components included in and excluded from this comprehensive definition is supplied in Supplemental Table 1.

### Estimating the added and free sugar content of foods and beverages

As food manufacturers in Australia are not currently required to display “added sugar” content in the NIP, we developed a method to estimate the “added sugar” content of packaged foods and beverages. This method was adapted from a previously published approach used to estimate the “added sugar” content of packaged and unpackaged foods as part of the last national dietary survey in Australia [[9], [21]]. However, as this method included several steps related to estimating “added sugars” in unpackaged foods, we were required to modify the approach to make it relevant to the packaged food supply only [9,21].

**FIGURE 2:**
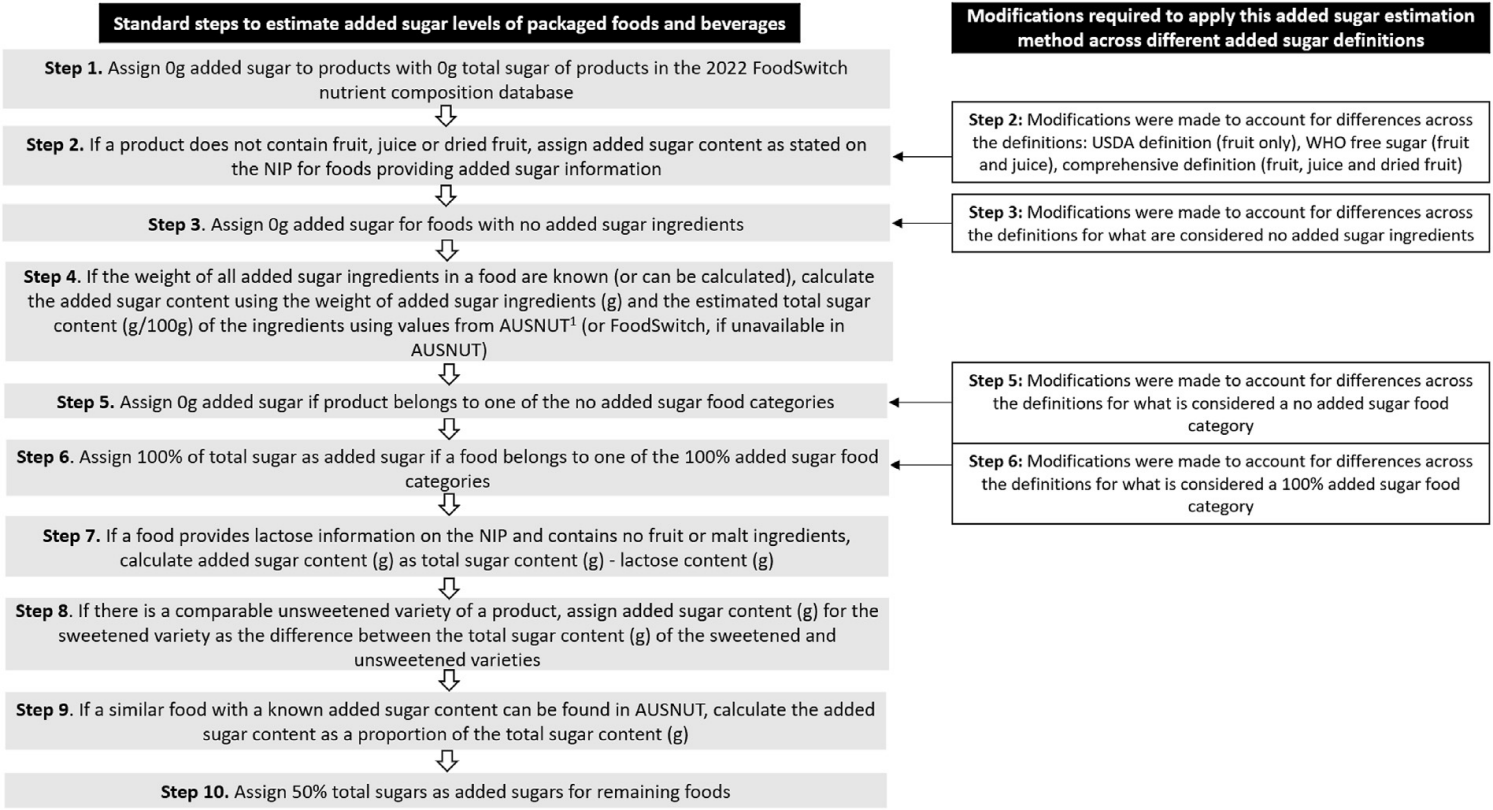
Outline of the methodology used to estimate the “added sugar” content of the packaged food supply in Australia. The methodology can be adapted to different added sugar definitions through modifying Steps 2, 3, 5 and 6 as indicated in the figure. ^1^AUSNUT, Australian Food and Nutrient database. AUSNUT is a nutrition database that contains nutrient values for 5740 generic foods and beverages [27] using information collected from a variety of sources including the nutrition information panel and ingredients list, data from laboratory analysis and data from international food composition databases [27, 28].

Our 10-step method utilizes information derived from the NIP and the ingredients list to identify products that contain specific "added sugars". This method is modifiable and can cater for a range of different “added sugar” definitions. A description of the main procedural steps is provided in Figure 2, and the approach used for specific definitions is displayed in Supplemental Figures 1–3. While the 10 steps are largely consistent across each of the definitions, there are differences across Steps 2, 3, 5, and 6. Step 2 can be modified to account for the types of sugars included in the definition. Step 3 can be modified to account for differences across the definitions for what is considered a “no added sugar” ingredient. Step 5 can be modified to account for differences across definitions for what is considered a “no added sugar” food category, and step 6 can be modified to account for differences across definitions for what is considered a 100% added sugar food category.

### Statistical analysis

Using summary statistics, we assessed the numbers and percentage of products containing “added sugars” according to each of the different “added sugar” definitions, overall, and by food category. We also calculated the mean ± standard deviation (SD) “added sugar” content overall and by each food category across each definition and compared this to the mean total sugar content. To assess the policy impact of a more inclusive “added sugar” definition, we compared the US FDA definition (least comprehensive) with the comprehensive definition to identify the most common foods and types of sugars that differ across the 2 definitions. All statistical analyses were conducted using Stata 15.1 (StataCorp).

## Results

### Included products

In total, 35,645 products were extracted from the FoodSwitch 2022 database. After removing ineligible and excluded products (*n* = 10,322), 25,323 products remained. These products were spread across 15 food categories, which ranged in size from 89 products in the egg and egg products category to 3,151 products in the dairy category.

### Number and proportion of products containing “added sugars” according to the added sugar definitions

Overall, the US FDA added sugar definition identified the lowest number and smallest proportion of products at 14,380 products (representing 56.8% of all products in the Australian food supply), followed by the WHO free sugar definition at 15,168 products (59.9%) and the comprehensive definition at 16,260 products (64.2%) (Table 1). Across all definitions, confectionary had the highest proportion of products containing “added sugars” (91.1% to 91.6%), followed by sugars, honey, and related products (89.5–90.5%) and convenience foods (76.8–85.1%) (Table 1).

**TABLE 1:** Numbers and percentage (%) of products containing added sugars according to each of the different added sugar definitions.

Food category	Total *N*	US FDA added sugar definition		WHO free sugar definition		Comprehensive definition	
		*n*	%	*n*	%	*n*	%
All categories combined	25,323	14,380	56.8	15,168	59.9	16,260	64.2
Bread and bakery products	3,000	1,958	65.3	1,945	64.8	1,979	66.0
Cereal and grain products	1,962	794	40.5	785	40.0	844	43.0
Confectionary	1,739	1,588	91.3	1,585	91.1	1,593	91.6
Convenience foods	1,848	1,420	76.8	1,491	80.7	1,573	85.1
Dairy	3,151	1,309	41.5	1,316	41.8	1,418	45.0
Edible oils and oil emulsions	530	3	0.6	3	0.6	4	0.8
Egg and egg products	89	2	2.2	2	2.2	2	2.2
Fish and fish products	806	327	40.6	333	41.3	350	43.3
Fruit, vegetables, nuts, and legumes	3,115	1,038	33.3	1,171	37.6	1,465	47.0
Meat and meat alternatives	1,702	1,238	72.7	1,210	71.1	1,268	74.5
Nonalcoholic beverages	2,125	967	45.5	1,511	71.1	1,543	72.6
Sauces, dressings, spreads, and dips	2,796	2,026	72.5	2,115	75.6	2,328	83.3
Snack foods	1,245	948	76.1	931	74.9	980	78.7
Special foods	741	336	45.3	346	46.7	484	65.3
Sugars, honey, and related products	474	426	89.9	424	89.5	429	90.5

### Estimated “added sugar” content according to added sugar definitions

Overall, the estimated mean (SD) “added sugar” content was lowest for the US FDA added sugar definition at 8.5 (16.5) g/100 g, followed by the WHO free sugar definition at 8.7 (16.4) g/100 g and the comprehensive definition at 9.6 (17.4) g/100 (Table 2). The mean “added sugar” content (g/100 g) across these 3 definitions was 3.0 to 4.1 g lower than the total sugar content (Table 2). Food categories with the highest mean “added sugar” content across all definitions included sugars, honey, and related products at 64.8 to 65.4 g/100 g, confectionary at 38.9 to 39.0 g/100 g and bread and bakery products at 12.4g/100 g (Table 2).

**TABLE 2:** Estimated mean “added sugar” content (g/100 g) according to each definition, by food category.

Food category	Total *N*	Estimated “added sugar” content, g/100 g (mean ± SD)			
		US FDA added sugar definition	WHO free sugar definition	Comprehensive definition	Total sugar
All categories combined	25,323	8.5 ± 16.5	8.7 ± 16.4	9.6 ± 17.4	12.6 ± 18.6
Bread and bakery products	3,000	12.4 ± 15.3	12.4 ± 15.3	12.4 ± 15.3	16.2 ± 16.2
Cereal and grain products	1,962	2.9 ± 6.2	3.0 ± 6.3	3.0 ± 6.2	5.3 ± 7.1
Confectionary	1,739	39.0 ± 20.1	38.9 ± 20.1	39.0 ± 20.0	44.7 ± 20.3
Convenience foods	1,848	1.2 ± 1.8	1.2 ± 1.8	1.2 ± 1.8	3.1 ± 3.0
Dairy	3,151	4.7 ± 8.0	4.7 ± 8.0	4.8 ± 7.9	7.9 ± 9.8
Edible oils and oil emulsions	530	0.0 ± 1.1	0.0 ± 1.1	0.0 ± 1.1	0.5 ± 2.2
Egg and egg products	89	0.0 ± 0.1	0.0 ± 0.1	0.0 ± 0.1	0.4 ± 0.4
Fish and fish products	806	1.1 ± 2.5	1.1 ± 2.5	1.1 ± 2.5	1.5 ± 2.4
Fruit, vegetables, nuts, and legumes	3,115	5.0 ± 12.4	5.1 ± 12.4	11.0 ± 20.3	14.3 ± 20.1
Meat and meat alternatives	1,702	1.4 ± 2.7	1.4 ± 2.7	1.4 ± 2.7	2.0 ± 2.8
Nonalcoholic beverages	2,125	3.4 ± 7.0	5.9 ± 7.1	5.9 ± 7.1	7.2 ± 10.5
Sauces, dressings, spreads, and dips	2,796	7.4 ± 11.6	7.4 ± 11.5	8.4 ± 12.6	11.9 ± 15.0
Snack foods	1,245	5.9 ± 8.0	6.0 ± 8.0	6.3 ± 8.3	10.7 ± 11.9
Special foods	741	2.2 ± 4.4	2.2 ± 4.4	3.3 ± 5.7	10.9 ± 12.0
Sugars, honey, and related products	474	64.8 ± 31.0	65.0 ± 31.0	65.4 ± 30.7	66.9 ± 30.2

### Key differences across the US FDA and comprehensive definition

When comparing the least and most comprehensive “added sugar” definitions (US FDA vs. the comprehensive definition), the comprehensive definition captured an additional 7.4% of products overall. However, a large variation by food category was observed, which ranged from a 0.0% difference for egg and egg products to a 27.1% difference for nonalcoholic beverages (Table 3). The most common foods that contributed to differences found between the 2 definitions for nonalcoholic beverages were fruit and vegetable juices, nectars, cordials, and herbal teas. These differences were mainly attributed to the different treatment of sugars for fruit and vegetable juices, fruit and vegetables purees, fruit nectars and pulps, and dried fruit across the 2 definitions. Similarly, the 20% difference in the proportions of products containing “added sugars” within the special foods category was driven by differential definition effects across baby and infant foods, toddler snacks, protein balls, diet, and protein bars (Table 3). The most common sugars that contributed to these differences included dried fruit; fruit and vegetable juice; and fruit and vegetables purees, pastes, and powders. Lastly, the most common foods that contributed to the 13.7% difference between the US FDA and comprehensive definitions were dried fruit and dried fruit products, jam, canned fruits and vegetables, pickled vegetables and dried vegetables (Table 3). These differences were attributed to the following sugars: dried fruits, fruit juices, powders and pulp, vegetable pastes, dehydrated/dried vegetables, vegetable powders, vegetable purees, and vegetable juices.

**TABLE 3:** Difference in the percentage of products containing added sugars (%) and difference in mean added sugars content (g/100 g) across the US FDA and comprehensive definition, ranked by food category.

Major food category	Difference in percentage of products containing added sugars (%)	Difference in the mean “added sugar” content (g/100 g)	Common foods driving the discordance between the US FDA and comprehensive definition	Common added sugars driving the discordance between the US FDA and comprehensive definition
Nonalcoholic beverages	27.1%	2.5	100% fruit juice, 100% vegetable juice, mixed fruit and vegetable juice, juice drinks, nectars, coconut water, aloe vera drinks, cordials, herbal tea, kombucha	Reconstituted fruit juice, fruit juice, fruit puree, fruit powder, vegetable juice, fruit pulp, dried vegetables, vegetable puree, coconut water, fruit nectar, dried fruit, fruit juice from concentrate
Special foods	20.0%	1.1	Protein balls, protein and diet bars, baby food (e.g., purees, cereal, porridge), toddler snacks	Dried fruit, fruit puree, fruit paste, dried fruit paste, fruit juice, fruit powder, vegetable juice, vegetable powder, vegetable paste, vegetable puree
Fruit, vegetables, nuts, and legumes	13.7%	6.0	Dried fruit, jam/marmalade, dried fruit bites and bars, candied fruit, fruit purees, quince paste, dried fruit and nut mixes, canned fruit, canned beans, canned vegetables, dried vegetables, pickled vegetables	Dried fruit, fruit juice concentrate, fruit juice, vegetable paste, dehydrated vegetables, vegetable powder, refined fruit juice, vegetable juice, vegetable puree, dried vegetables, fruit powder, fruit pulp
Sauces, dressings, spreads, and dips	10.8%	1.0	Vinegar including balsamic, dips, BBQ and tomato sauce, apple sauce, relish, chutney, tapenades, tomato-based pasta sauces, tamarind paste, tomato paste, curry paste	Vegetable puree, vegetable paste, reconstituted vegetables, dehydrated vegetables, dried vegetables, vegetable juice, vegetable concentrate, vegetable pulp, fruit juice concentrate, vegetable powder, fruit puree, fruit juice, fruit paste, dried fruit
Convenience foods	8.3%	0.0	Ready meals (e.g., lasagna, chili con carne, curries), vegetable soups, vegetable pastries, salads	Fruit juice, dried fruit, fruit juice concentrate, vegetable puree, vegetable paste, vegetable juice, fruit paste, dried/dehydrated vegetables, vegetable powder
Dairy	3.5%	0.1	Flavored and unflavored dairy yogurt, flavored milk, coconut milk yogurt, dairy free cheese, ice-cream sticks, drinking yogurts	Fruit juice concentrate, fruit puree, fruit pulp, fruit concentrate, vegetable concentrate, fruit juice, reconstituted fruit juice, fruit paste
Fish and fish products	2.7%	0.0	Flavored canned fish (e.g., tuna, sardines), other flavored canned seafood (e.g., mussels)	Vegetable paste, fruit juice powder, fruit juice
Snack foods	2.6%	0.4	Tuna snack pots, muesli bars, vegetable and legume-based snacks, corn chips	Fruit powder, fruit juice, vegetable paste, vegetable powder, fruit paste
Cereal and grain products	2.5%	0.1	Flavored oats and porridge, muesli, flavored rice, ravioli, gnocchi	Fruit juice, dried vegetables, dried fruit, vegetable paste, vegetable powders
Meat and meat alternatives	1.8%	0.0	Sausages, burger patties, meat pies, meat-free sausages, meat-free burger patties	Dehydrated vegetables, vegetable puree, vegetable paste, vegetable powder
Bread and bakery products	0.7%	0.0	Sweet biscuits (cookies), fruit and nut biscuits, fruit bread, cake/muffin mixes	Dried fruit, vegetable puree, fruit juice
Sugars, honey, and related products	0.6%	0.6	Flavored honey, sweet, flavored dessert sauce (e.g., strawberry topping)	Fruit puree, dried fruit
Confectionary	0.3%	0.0	Chocolate with fruit	Dried fruit
Edible oils and oil emulsions	0.2%	0.0	Garlic butter spread	Fruit juice
Egg and egg products	0.0%	0.0	n/a	n/a

There was also substantial variation in the estimated “added sugar” content according to the US FDA and comprehensive definitions. Although the overall difference was 1.1 g/100 g, the differences across food categories ranged from 0.0 g/100 g (i.e., no difference) for 7 food categories up to 6.0 g for the fruit, vegetables, nuts, and legumes category. The latter was primarily driven by fruit products and jams (Table 3). The next largest difference was for the nonalcoholic beverages category, where the 2.5 g/100 g difference was largely driven by fruit and vegetable juices, cordials, flavored water, and energy drinks. Lastly, there was a 1.1 g/100 g difference for the special foods category, which was driven by baby and infant foods, foods for special dietary use, and sports products.

## Discussion

This study applied a 10-step methodology to estimate the “added sugar” content of packaged foods to illustrate the practical implications of applying 3 different definitions for “added sugars” across >25,000 Australian packaged foods. We found that across all definitions, “added sugars” were in approximately 60% of all products, albeit with some variation according to different definitions and food categories. For all 3 definitions, meaningful differences were observed in outcomes when assessing “added sugar” and total sugar, highlighting the value of both metrics for inclusion in the NIP. Lastly, nonalcoholic beverages, special foods and fruit, vegetables, nuts, and legumes were most commonly impacted by the application of different definitions—with the comprehensive definition, that includes all “added” and “free” sugars linked to poor health comes—capturing more products due to the definition encompassing a wider range of fruit and vegetable sugars including purees, pastes, juices, and pulps.

A key finding from this study was the varying impact of the “added sugar” definitions in terms of number of impacted products and the estimated “added sugar” content. While the differences were small across most categories, some categories were greatly impacted under the most comprehensive added sugar definition, including fruit; vegetables; nut and legumes (dried fruit, jam, canned fruits and vegetables, pickled vegetables, and dried vegetables); special foods (baby and toddler foods, protein and diet products); and nonalcoholic beverages (fruit and vegetable juice, nectars, cordials and herbal tea). The primary reason for these differences is attributable to the treatment of dried fruit and processed fruit and vegetable sugars in the form of pastes, purees, and powders in the comprehensive definition. The inclusion of these fruit and vegetable sugars is based on the evidence for the high concentration of sugars and frequent use as a sweetening agent in food [16]. A precautionary approach to dried fruit is also recommended given the potential dental harms due to the stickiness on the teeth and the fact that current dietary guidance for dried fruit recommends limiting consumption [16, 22, 23]. If a regulatory definition for “added sugars” does not capture these fruit sugars, a substantial proportion of the Australian food supply, particularly certain food categories where these sugars are prominent, could be exempt from having to display “added sugars.” This is a concern as inclusion of these sugars is needed to ensure that the “added sugar” content adequately reflects the high sugar content of dried fruit and processed fruits and vegetables.

Another key finding from this paper is that ∼60% of packaged foods in Australia contained “added sugars.” This high prevalence of “added sugars” in the food supply is consistent with prior literature globally including from studies conducted in Turkey [24] and Canada [25], which found “added sugars” in 86.5% and 66% of all packaged foods, respectively. The frequent use of “added sugars” especially in certain food categories, such as confectionary, convenience foods, snack foods and sauces, dressings, spreads and dips, highlight the need for more nutrition policies in Australia and globally that target a reduction in “added sugars.” One way Australia is currently working to achieve this is through revising a regulatory definition for “added sugars” to inform changes pertaining to “no added sugar” claims [14]. This definition may also inform the introduction of mandatory “added sugar” labeling [15] and other future policy areas including modifications to the Health Star Rating system. To adequately communicate with the public about the presence and quantity of harmful sugars in excess, it is crucial to develop a definition that consumers can understand and that encompasses all types of sugars that have been linked to poor health outcomes. It is also important to develop a definition that is future-proofed by minimizing loopholes that could be exploited by food manufacturers by simply replacing one form of “added sugar” with another ingredient that has similar risks to health. This study suggests that key sugars that should be included but that are missing from some existing definitions (including the US FDA and WHO definitions) are dried fruits and processed fruit and vegetable sugars in the form of powders, pastes, pulps, and purees. The exclusion of these sugars is highly relevant to “no added sugar” claims. If they are not included within a regulatory definition, this could potentially mislead consumers by allowing products containing sugars that have been linked to poor harmful outcomes, such as processed fruit sugars, to display “no added sugar” claims and declare a low “added sugar” content in the NIP. Additionally, as claims are used as a powerful marketing tool [26], lack of a comprehensive definition could also encourage food manufacturers to substitute one form of “added sugars” with other sugars that are excluded from the definition for example, dried fruit, to make a “no added sugar” claim while retaining a product’s sweet flavor profile.

To conduct this study, we developed and presented a 10-step method for generating estimates for “added sugars.” Not only does this method rely on information readily available on packaged foods through the ingredient list and NIP, we have shown through this study that this method can be adapted for different definitions of “added sugars” by making small changes to 4 of the 10 steps. Given this method uses readily available information, it could be adopted by researchers and policymakers globally as a tool to estimate the “added sugar” content of products available in the market and to monitor changes in levels of “added sugars” in the food supply. This would be particularly important for estimating “added sugars” in countries that do not have mandatory labeling of “added sugars” and could help to drive further research in this space to better understand the presence of “added sugars” in packaged foods globally and the potential need for further regulation in this space.

The implementation of “added sugar” labeling will require the food industry to calculate the amount of “added sugar” in their products, which can be done through the use of recipes [27]. For example, in the United States, where “added sugar” labeling has been adopted, manufacturers are required to keep paperwork for inspection to confirm the accuracy of their calculations for the purposes of enforcement [27]. Assessing industry compliance to added sugar labeling and the impact of labeling to changes in the “added sugars” content of the food supply will be important to monitor over time. It is also important for policymakers to monitor other areas of the food supply that could be impacted by changes to “added sugar” labeling. This could potentially include widespread full or partial substitution of “added sugars” with nonnutritive sweeteners [28]. Such substitutions from “added sugars” to artificial sweeteners was observed in Chile after the introduction of a food labeling policy that requires packaged foods and beverages to carry a front-of-pack “high in sugars” warning label if they exceed limits for sugar [29]. Replacing “added” or “free” sugars with artificial sweeteners is not recommended by the WHO for improving weight control or long-term health outcomes based on a review of the evidence regarding the health risks associated consumption of artificial sweeteners [30]. Instead, the WHO recommends that “added sugars” should be reduced not replaced across the whole of the diet to improve health [30]. Such an approach should be taken by food manufacturers when they are reformulating their product lines to reduce the “added sugar” content.

A key strength of this study is the use of a large sample of packaged food and beverage products representative of the contemporary Australian food supply. Our application of 3 “added sugar” definitions ranging in comprehensiveness allowed for realistic and practical implications of using different “added sugar” definitions across the Australian food supply and identified the key foods and sugars driving these differences. We have proposed an objective and systematic approach for estimating the “added sugar” content of the food supply that relies on a readily available information from packaged foods and that can be adapted for a range of “added sugar” definitions.

Some limitations should be mentioned. Our methodology for estimating “added sugar” does not account for sugars produced during processing methods, such as hydrolysis and fermentation. If a regulatory definition for Australia includes these processing sugars, the proposed method would need to be updated to include an additional step that accounts for these sugars. Moreover, given that our study analyzed packaged foods available for sale in Australia, the results may not be generalizable to all countries globally, especially countries that utilize different forms of sugars and have different types of products available for sale.

In conclusion, “added sugars” were found in approximately 60% of all foods, with levels highest in confectionary, sugars, honey, and related products and convenience foods. Levels of “added sugars” in nonalcoholic beverages, special foods and fruit, vegetables, nuts, and legumes were most frequently impacted by the differences across the definitions in the treatment of dried fruits and processed fruit and vegetable sugars. Australia should consider adopting a comprehensive regulatory definition for “added sugars” that encompasses all sugars that are linked to poor health outcomes and that minimizes the substitution of "added sugars" with other ingredients with comparable health risks.

## Conflict of interest

The authors report no conflicts of interest.

## Funding

None.

## Data availability

Data described in the manuscript were used under license for the current study. Data may be made available upon request to FoodSwitch Manager, Fraser Taylor.
