# Supplementary material for: Assessing the Policy Implications of Different Definitions for Added Sugars: An Analysis Across the Australian Packaged Food and Beverage Supply
Source: Curr Dev Nutr. 2024 Feb 20;8(2):102058. doi: 10.1016/j.cdnut.2023.102058 (PMC10926138; doi:10.1016/j.cdnut.2023.102058)
Supplement: Multimedia component 1 [file mmc1.docx]

Assessing the policy implications of different definitions for 'added sugars': An analysis across the Australian packaged food and beverage supply

Daisy H Coyle

**Supplementary Table 1.** List of food components proposed to be included and excluded in the comprehensive definition of added sugars

| **Included** | **Excluded** |
| --- | --- |
| - Sugars in whatever form and from whatever source (e.g., cane sugar, beet sugar, white sugar, brown sugar, granulated sugar, icing sugar, fruit sugar, invert sugar) - Monosaccharides and disaccharides isolated from their original food sources and added as an ingredient to foods or drinks (e.g., lactose – including lactose in whey powder, galactose, fructose) - All sugars naturally present in processed fruit and vegetables (blended, juices, pastes, purée, powdered, concentrates, nectars) when sugars are no longer in their natural cellular structure - Concentrated fruit or vegetable juice or deionised fruit or vegetable juice - Dried fruits - Sugars naturally present in syrups (e.g., maple syrup, golden syrup, high-fructose corn syrup, glucose syrup, agave syrup), honeys, molasses, treacle, malt and malt extract, starch hydrolysate, maltodextrin and similar products | - Lactose and galactose when naturally present in milk and dairy or dairy-based products All sugars naturally present in fresh and some processed (stewed, canned and frozen) fruit and vegetables (including beans) when sugars remain in their natural cellular structure (e.g. the intact fruit component (whole or pieces) of tinned fruit would not be added sugars, but any sugars added in the form of syrup to that product would be) All sugars naturally present in cereal grains including rice, pasta and flour regardless of processing (other than cereal-based drinks) All sugars naturally present in nuts and seeds regardless of processing Sugar substitutes that do not contains sugars, such as polyols (sorbitol) and other non-nutritive sweeteners |

| **Step 1.** Assign 0g added sugar to foods with 0g total sugar of products in the 2022 FoodSwitch nutrient composition database (n = 1,429) |
| --- |

| **Step 2**. If product does not contain fruit ingredients, assign added sugar content as stated on the NIP for foods providing added sugar information (n = 1,213) |
| --- |

| **Step 3**. Assign 0g added sugar for foods with no added sugar ingredients i.e., if product does not contain any of the added sugar ingredients (n = ,7074) |
| --- |

| **Step 4.** If the weight of all added sugar ingredients in a food are known (or can be calculated), calculate the added sugar content using the weight of added sugar ingredients (g) and the estimated total sugar content (g/100g) of the ingredients using values from AUSNUT^1^ (or FoodSwitch, if unavailable in AUSNUT) (n = 222) |
| --- |

| **Step 5.** Assign 0g added sugar if product belongs to one of the no added sugar food categories  (n = 360)  No added sugar food categories include: *100% fruit/vegetable juices, spices and herbs, fats and oils, all plain cereal grains, pastas, rice, and flours, eggs, fresh fruit, fresh vegetables, fresh meat, fresh seafood, and tofu, fruits canned in 100% fruit juice or liquid sweetened with nonnutritive sweeteners only, intensely sweetened jam and beverage base (without added sugar), legumes (fresh, dried, and/or processed, except sweetened varieties), mixed meat dishes with no added sugar, unsweetened alcoholic beverages, unsweetened coffees and tea, non-sugar-sweetened milk and buttermilk, non-sugar-sweetened dairy products (including yoghurts sweetened with nonnutritive sweeteners), nuts, coconut, and seeds (except sweetened varieties), oats (and porridge) with no added sugar, plain pastries without filling (such as chocolate, dried fruit, and/or nuts), plain breads (except gluten-free), plain breads (except gluten-free) and other plain bakery products e.g. English muffins, bagels, unsweetened dried fruits* |
| --- |

| **Step 6.** Assign 100% of total sugar as added sugar if a food belongs to one of the 100% added sugar food categories (n = 5,499)  100% added sugar food categories include: *All confectionery except those containing dairy products, breakfast cereals and cereal bars without fruits, chocolate, dairy, or milk solids, coffee and beverage base with no milk solids, crumbed/battered meat and seafood, processed meats, regular soft drinks, sport drinks, flavoured water, and non-fruit-based energy drink, savoury biscuits, sweet biscuits, cakes and buns, donuts, and batter-based products that do not contain fruit, chocolate, or dairy products, soy and other plant-based beverages and yoghurts without added fruits, stock powder, sugar and syrup.* |
| --- |

| **Step 7.** If a food provides lactose information on the NIP and contains no fruit or malt ingredients, calculate added sugar content (g) as total sugar content (g) - lactose content (g)  (n = 56) |
| --- |

| **Step 8.** If there is a comparable unsweetened variety of a product, assign added sugar content (g) for the sweetened variety as the difference between the total sugar content (g) of the sweetened and unsweetened varieties (n = 661) |
| --- |

| **Step 9.**  If a similar food with a known added sugar content can be found in AUSNUT, calculate the added sugar content as a proportion of the total sugar content (g) (n = 6,092) |
| --- |

| **Step 10.** Assign 50% total sugars as added sugars for remaining foods (n = 2,717) |
| --- |

**Supplementary Figure 1. Outline of the methodology used to estimate the added sugar content of the packaged food supply in Australia according to the US FDA definition for added sugars.** ^1^AUSNUT, Australian Food and Nutrient database. AUSNUT is a nutrition database that contains nutrient values for 5740 generic foods and beverages [21] using information collected from a variety of sources including the NIP and ingredients list, data from laboratory analysis and data from international food composition databases [21, 22]. N indicates the number of products assigned at each step.

| **Step 1.** Assign 0g added sugar to foods with 0g total sugar of products in the 2022 FoodSwitch nutrient composition database (n =1,429) |
| --- |

| **Step 2**. If product does not contain fruit or juice ingredients, assign added sugar content as stated on the NIP for foods providing added sugar information (n = 1,050) |
| --- |

| **Step 3**. Assign 0g added sugar for foods with no added sugar ingredients i.e., if product does not contain any of the added sugar ingredients (n = 6,933) |
| --- |

| **Step 4.** If the weight of all added sugar ingredients in a food are known (or can be calculated), calculate the added sugar content using the weight of added sugar ingredients (g) and the estimated total sugar content (g/100g) of the ingredients using values from AUSNUT^1^ (or FoodSwitch, if unavailable in AUSNUT) (n = 323) |
| --- |

| **Step 5.** Assign 0g added sugar if product belongs to one of the no added sugar food categories  (n = 360)  No added sugar food categories include: *Spices and herbs, fats and oils, all plain cereal grains, pastas, rice, and flours, eggs, fresh fruit, fresh vegetables, fresh meat, fresh seafood, and tofu, fruits canned in 100% fruit juice or liquid sweetened with nonnutritive sweeteners only, intensely sweetened jam and beverage base (without added sugar), legumes (fresh, dried, and/or processed, except sweetened varieties), mixed meat dishes with no added sugar, unsweetened alcoholic beverages, unsweetened coffees and tea, non-sugar-sweetened milk and buttermilk, non-sugar-sweetened dairy products (including yoghurts sweetened with nonnutritive sweeteners), nuts, coconut, and seeds (except sweetened varieties), oats (and porridge) with no added sugar, plain pastries without filling (such as chocolate, dried fruit, and/or nuts), plain breads (except gluten-free) and other plain bakery products e.g. English muffins, bagel, unsweetened dried fruits.* |
| --- |

| **Step 6.** Assign 100% of total sugar as added sugar if a food belongs to one of the 100% added sugar food categories (n = 5,510)  100% added sugar food categories include: *All confectionery except those containing dairy products, breakfast cereals and cereal bars without fruits, chocolate, dairy, or milk solids, coffee and beverage base with no milk solids, crumbed/battered meat and seafood, processed meats, regular soft drinks, sport drinks, flavoured water, and non-fruit-based energy drink, savoury biscuits, sweet biscuits, cakes and buns, donuts, and batter-based products that do not contain fruit, chocolate, or dairy products, soy and other plant-based beverages and yoghurts without added fruits, stock powder, sugar and syrup, honey and fruit and vegetable juices.* |
| --- |

| **Step 7.** If a food provides lactose information on the NIP and contains no fruit or malt ingredients, calculate added sugar content (g) as total sugar content (g) - lactose content (g)  (n = 51) |
| --- |

| **Step 8.** If there is a comparable unsweetened variety of a product, assign added sugar content (g) for the sweetened variety as the difference between the total sugar content (g) of the sweetened and unsweetened varieties (n = 728) |
| --- |

| **Step 9.**  If a similar food with a known added sugar content can be found in AUSNUT, calculate the added sugar content as a proportion of the total sugar content (g) (n = 6,165) |
| --- |

| **Step 10.** Assign 50% total sugars as added sugars for remaining foods (n = 2,774) |
| --- |

**Supplementary Figure 2. Outline of the methodology used to estimate the added sugar content of the packaged food supply in Australia according to the WHO definition for free sugars.** ^1^AUSNUT, Australian Food and Nutrient database. AUSNUT is a nutrition database that contains nutrient values for 5740 generic foods and beverages [21] using information collected from a variety of sources including the NIP and ingredients list, data from laboratory analysis and data from international food composition databases [21, 22].

| **Step 1.** Assign 0g added sugar to foods with 0g total sugar of products in the 2022 FoodSwitch nutrient composition database (n = 1,429) |
| --- |

| **Step 2**. If product does not contain fruit, juice or dried fruit ingredients, assign added sugar content as stated on the NIP for foods providing added sugar information (n = 687) |
| --- |

| **Step 3**. Assign 0g added sugar for foods with no added sugar ingredients i.e., if product does not contain any of the added sugar ingredients (n = 6,557) |
| --- |

| **Step 4.** If the weight of all added sugar ingredients in a food are known (or can be calculated), calculate the added sugar content using the weight of added sugar ingredients (g) and the estimated total sugar content (g/100g) of the ingredients using values from AUSNUT^1^ (or FoodSwitch, if unavailable in AUSNUT) (n = 440) |
| --- |

| **Step 5.** Assign 0g added sugar if product belongs to one of the no added sugar food categories  (n = 362)  No added sugar food categories include: *Spices and herbs, fats and oils, all plain cereal grains, pastas, rice, and flours, eggs, fresh fruit, fresh vegetables, fresh meat, fresh seafood, and tofu, fruits canned in 100% fruit juice or liquid sweetened with nonnutritive sweeteners only, intensely sweetened jam and beverage base (without added sugar), legumes (fresh, dried, and/or processed, except sweetened varieties), mixed meat dishes with no added sugar, unsweetened alcoholic beverages, unsweetened coffees and tea, non-sugar-sweetened milk and buttermilk, non-sugar-sweetened dairy products (including yoghurts sweetened with nonnutritive sweeteners), nuts, coconut, and seeds (except sweetened varieties), oats (and porridge) with no added sugar, plain pastries without filling (such as chocolate, dried fruit, and/or nuts), plain breads (except gluten-free) and other plain bakery products e.g. English muffins, bagels.* |
| --- |

| **Step 6.** Assign 100% of total sugar as added sugar if a food belongs to one of the 100% added sugar food categories (n = 5,655)  100% added sugar food categories include: *All confectionery except those containing dairy products, breakfast cereals and cereal bars without fruits, chocolate, dairy, or milk solids, coffee and beverage base with no milk solids, crumbed/battered meat and seafood, processed meats, regular soft drinks, sport drinks, flavoured water, and non-fruit-based energy drink, savoury biscuits, sweet biscuits, cakes and buns, donuts, and batter-based products that do not contain fruit, chocolate, or dairy products, soy and other plant-based beverages and yoghurts without added fruits, stock powder, sugar and syrup, honey, fruit and vegetable juice, dried fruit.* |
| --- |

| **Step 7.** If a food provides lactose information on the NIP and contains no fruit or malt ingredients, calculate added sugar content (g) as total sugar content (g) - lactose content (g)  (n = 56) |
| --- |

| **Step 8.** If there is a comparable unsweetened variety of a product, assign added sugar content (g) for the sweetened variety as the difference between the total sugar content (g) of the sweetened and unsweetened varieties (n = 777) |
| --- |

| **Step 9.**  If a similar food with a known added sugar content can be found in AUSNUT, calculate the added sugar content as a proportion of the total sugar content (g) (n = 6,474) |
| --- |

| **Step 10.** Assign 50% total sugars as added sugars for remaining foods (n = 2,886) |
| --- |

**Supplementary Figure 3. Outline of the methodology used to estimate the added sugar content of the packaged food supply in Australia according to the public health definition for free sugars.**

^1^AUSNUT, Australian Food and Nutrient database. AUSNUT is a nutrition database that contains nutrient values for 5740 generic foods and beverages [21] using information collected from a variety of sources including the NIP and ingredients list, data from laboratory analysis and data from international food composition databases [21, 22].
